# Secret Preparations

**Published:** 1891-07

**Authors:** S. B. Palmer

**Affiliations:** Syracuse, N. Y.


### SECRET PREPARATIONS.

BY DR. S. B. PALMER, SYRACUSE, N. Y.

THE numerous venders in our profession of secret preparations would seem to indicate that there must be purchasers, and that dentistry is on a retrograde movement. Surely we can hardly imagine that individuals are so highly interested in suffering humanity as to give their time gratuitously to this work. I was requested to try an obtundent for sensitive dentine and report my opinion. The assurance was given that the compound contained no "arsenic, strychnine, or any drug that could destroy the nerve." Having made the examination I feel it my duty to give the conclusions to the profession as well as to the discoverer. The following is the result of the trial: Three drops of the obtundent were added to one ounce of water. The test gave a strong acid reaction. The next was for a metal: the electrodes from a battery were attached to two plates of gold-foil, and before the gold was lowered half-way its length into the fluid that portion at the negative pole became coated with a metal of a dark lead color. In the report made to me Dr. James Truman's name was mentioned as one who by letter abruptly refused to make a trial of the obtundent. I sent to him the gold electrodes and test-papers as evidences that his position in refusing secret compounds was the correct one.[1] To give the name of the dentist referred to might be construed as a breach of confidence.

The only true method to adopt in cases of this nature is to educate dentists to refuse the use of all preparations in cases where there could be a possibility of harm resulting. No one can tell the final results of an unknown metal in its action upon vital dentine. We know something of the effects of arsenic when used as an obtundent; other metals with combinations may be equally harmful. When escharotics are applied to muscle, sloughing occurs, which might be dangerous if received into the circulation. Dentine being a solid, retains all that cannot be washed from the surface.

There are two distinct methods in the action of an obtundent. One is suspended animation as produced by hot air, cold spray, and by various other temporary agents; the other is devitalization of

---

[1] Dr. Palmer sends with his paper two gold electrodes and several test-papers as evidence of his statements. One of the electrodes sent indicates a deposit of a dark lead color, and the test-papers give marked evidence of acid reaction.—ED.

sensibility, as by arsenic, mercury, acids, etc. In dentine the dead portion must remain in contact with the living. When a metallic acid is introduced the acid is soon neutralized by the lime to render its further action harmless. This sets the atoms of the metal free to combine with the vital elements of the organic structure, and the process of devitalization continues, possibly for years. We have a correspondence in the effect of a few drops of acid upon the pages of a book. History and observations fail to limit its action; in fact, the destruction we are told does not stop, the leaves crumble and fall away in dust; so "dry-rot" in timber in time destroys wood.

The science of this vital action seems reasonable, as, I believe organic bodies are built from within by currents of a negative or alkaline nature; externally are the opposite or acid currents. Let us apply this statement to a condition of the teeth occasionally seen, which have been worn to the gums, the pulp having receded and protected itself by secondary deposits. At no time have the pulps been in an abnormal condition, consequently the organs of nutrition did the work. If the destruction of tissue be from cavities or by lodgement of food allowed to ferment, no such resistance would occur, because the potentials would be changed. I believe both in the polarity and potential of vital currents, as in the electroplating, where the changes can be witnessed.

The absorption of the roots of deciduous teeth afford still another illustration. By reversing the nutrient current the root is absorbed by a process opposed to that which built it up. In this case two or more roots only are subject to the resorptive process. In case of gestation the whole secretions become abnormal, and all the teeth are effected in that a portion is taken from the lime elements. There is an uncultivated field in this direction, yet the promise is great, and the way seems much less difficult than the solving of the problem which has so recently been done. Why do teeth decay?

